# Whole Exome Sequencing Enhanced Imputation Identifies 85 Metabolite Associations in the Alpine CHRIS Cohort

**DOI:** 10.3390/metabo12070604

**Published:** 2022-06-29

**Authors:** Eva König, Johannes Rainer, Vinicius Verri Hernandes, Giuseppe Paglia, Fabiola Del Greco M., Daniele Bottigliengo, Xianyong Yin, Lap Sum Chan, Alexander Teumer, Peter P. Pramstaller, Adam E. Locke, Christian Fuchsberger

**Affiliations:** 1 Institute for Biomedicine (Affiliated Institute of the University of Lübeck), Eurac Research, 39100 Bolzano, Italy; eva.koenig@eurac.edu (E.K.); johannes.rainer@eurac.edu (J.R.); viniciusveri@gmail.com (V.V.H.); fabiola.delgreco@eurac.edu (F.D.G.M.); daniele.bottigliengo@eurac.edu (D.B.); peter.pramstaller@eurac.edu (P.P.P.); 2 School of Medicine and Surgery, University of Milano-Bicocca, 20854 Vedano al Lambro, Italy; giuseppe.paglia@unimib.it; 3 Department of Biostatistics, Center for Statistical Genetics, University of Michigan School of Public Health, Ann Arbor, MI 48109, USA; xyyin@umich.edu (X.Y.); lapsum@umich.edu (L.S.C.); 4 Institute for Community Medicine, University Medicine Greifswald, 17475 Greifswald, Germany; ateumer@uni-greifswald.de; 5 German Centre for Cardiovascular Research (DZHK), Partner Site Greifswald, 17475 Greifswald, Germany; 6 Department of Neurology, Central Hospital, 39100 Bolzano, Italy; 7 Department of Medicine, McDonnell Genome Institute, Washington University School of Medicine, St. Louis, MO 63108, USA; adam.locke@regeneron.com

**Keywords:** GWAS, ExWAS, association study, whole-exome sequencing, imputation, metabolomics

## Abstract

Metabolites are intermediates or end products of biochemical processes involved in both health and disease. Here, we take advantage of the well-characterized Cooperative Health Research in South Tyrol (CHRIS) study to perform an exome-wide association study (ExWAS) on absolute concentrations of 175 metabolites in 3294 individuals. To increase power, we imputed the identified variants into an additional 2211 genotyped individuals of CHRIS. In the resulting dataset of 5505 individuals, we identified 85 single-variant genetic associations, of which 39 have not been reported previously. Fifteen associations emerged at ten variants with >5-fold enrichment in CHRIS compared to non-Finnish Europeans reported in the gnomAD database. For example, the CHRIS-enriched *ETFDH* stop gain variant p.Trp286Ter (rs1235904433-hexanoylcarnitine) and the *MCCC2* stop lost variant p.Ter564GlnextTer3 (rs751970792-carnitine) have been found in patients with glutaric acidemia type II and 3-methylcrotonylglycinuria, respectively, but the loci have not been associated with the respective metabolites in a genome-wide association study (GWAS) previously. We further identified three gene-trait associations, where multiple rare variants contribute to the signal. These results not only provide further evidence for previously described associations, but also describe novel genes and mechanisms for diseases and disease-related traits.

## 1. Introduction

The human metabolism comprises the entire set of biochemical processes that govern the life of cells. Metabolites are small molecules that represent intermediate or end products of cellular metabolism. Their concentrations in blood are influenced by genetics, but also environmental factors, such as lifestyle and dietary habits. Metabolite levels can provide insights into the physiological state of the body. Dysregulations often indicate critical physiological states or presence of metabolic diseases, such as diabetes, chronic kidney disease or inherited metabolic disorders, such as phenylketonuria and medium-chain acyl-CoA dehydrogenase deficiency [1,2,3].

In the last decade, over 240,000 metabolite-variant associations have been discovered, many of which account for up to 50% of the total variance in metabolite levels [4,5,6,7,8]. Most of these studies tested associations between metabolite levels and predominantly common variants (minor allele frequency (MAF) > 5%). However, there are compelling reasons to focus on variations in the protein-coding sequence. First, coding variants are enriched for impact on molecular function and support more direct biological interpretation than associations within a non-coding sequence. Second, the functional annotation of coding variants allows discovery efforts to benefit from the improved power offered by the aggregation of rare alleles presumed to exert broadly similar molecular effects through gene-based aggregation tests, which enables the direct testing of gene-phenotype associations. Finally, the development of efficient tools to interrogate coding variation, through whole-exome sequencing (WES) or custom array genotyping, has made it practical to investigate low-frequency (MAF 1–5%) and rare (MAF < 1%) variants in large sample sizes. Recently, Barton et al. introduced a cost-efficient approach to characterize the coding genome by imputing variants of 49,960 exome-sequenced individuals from the UK Biobank into the remainder of the cohort, exploiting the extensive haplotype sharing within the UK Biobank [9].

Here, we follow a similar approach, in which we exome sequenced 3294 participants of the Cooperative Health Research in South Tyrol (CHRIS) study followed by an imputation of those variants into the remaining 2211 individuals for whom only genotype data was available. The CHRIS study is a population-based longitudinal study to investigate the genetic and molecular basis of age-related common chronic conditions and their interaction with environment in North Italy [10]. We then performed an exome-wide association study (ExWAS) of 175 metabolites in the full set of 5505 WES-imputed individuals. We identified 85 significant single variant-trait associations for 40 metabolites. When testing the aggregate effects of coding variants in genes, we identified three additional gene-metabolite associations. Among our significant findings, 15 rare associations are more than five times more common in CHRIS than in non-Finnish Europeans in gnomAD. This demonstrates the value of both exome sequencing followed by imputation for ExWAS and the value of exploring diverse population cohorts, even if they might be smaller compared to others, for the identification of novel variant-trait associations in the age of biobank-level studies [11].

## 2. Results

### 2.1. Study Design and Genotype Data

**Figure 1:**
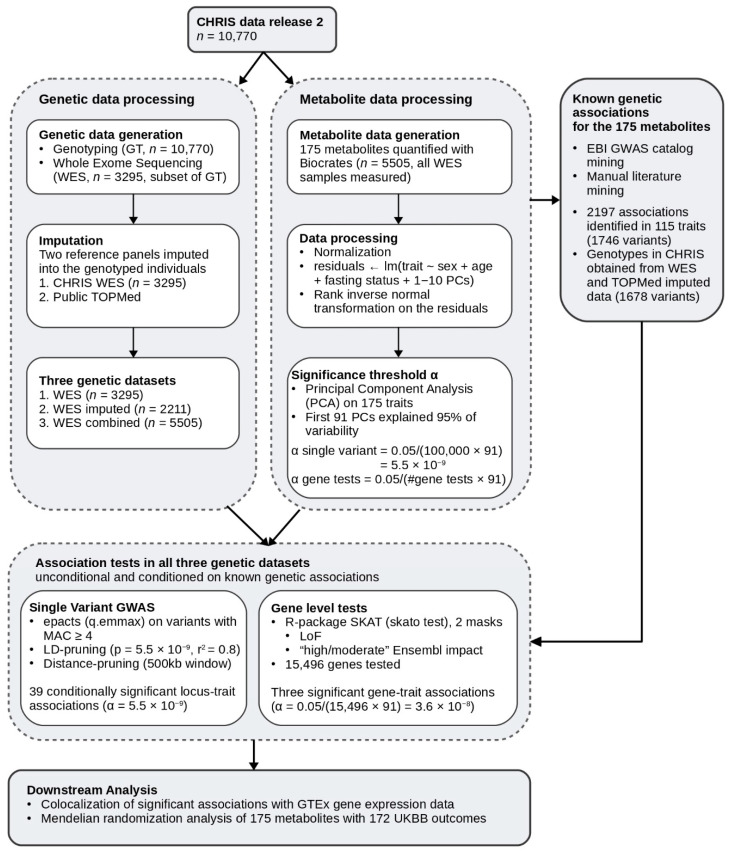
Flowchart of the study methods and summary of results.

The study design is summarized in Figure 1. In brief, we assayed 175 metabolites in 5505 CHRIS participants of which 3294 have been whole-exome sequenced. To increase power, we imputed the 1,023,678 identified sequence variants into an additional 2211 genotyped CHRIS participants. Overall, variants were imputed with a mean estimated imputation accuracy (rsq) of 0.757 (median = 0.815, sd = 0.211). Imputed variants with rsq < 0.3 were discarded, retaining 95.3% of all imputed variants, resulting in a mean and median rsq of 0.788 and 0.825 for the retained variants, respectively.

**Figure 2:**
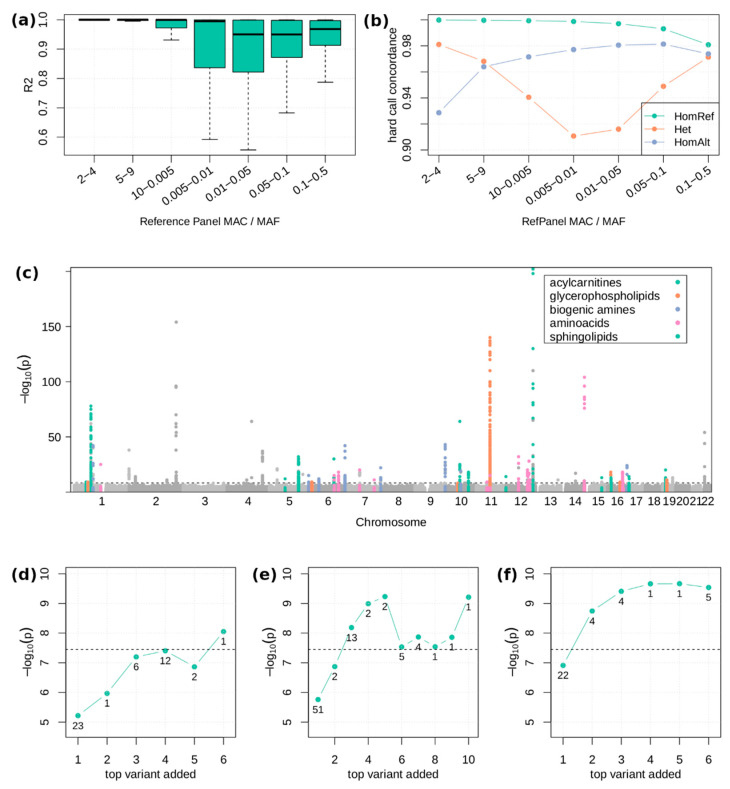
Main results of the metabolite ExWAS. (**a**) Squared correlation of imputed dosages and sequenced genotypes in 181 validation samples split into bins based on MAC or MAF in the reference panel. Number of variants included in the bins from left to right: 2–4: 1851; 5–9: 4412; 10–0.005 = 24,868; 0.005–0.01 = 24,232; 0.01–0.05 = 53,488; 0.05–0.1 = 19,669; 0.1–0.5 = 57,563. (**b**) Concordance of imputed and sequenced hard calls in 181 validation samples split into bins based on MAC and MAF in the reference panel. (**c**) Manhattan plot of the single-variant associations of all 175 traits. The 85 significant associations listed in Table S4 are highlighted and colored by metabolite class. The dashed horizontal line indicates the significance threshold of 5.5 × 10^−9^. (**d**–**f**) −log10 *p*-value of the skato gene test, adding the variants constituting the gene test iteratively for tryptophan—*TDO2* (**d**), sphingomyeline C18:0—*CERS4* (**e**), and carnitine—*SLC22A5* (**f**). In each step i on the *x*-axis, the gene test is computed using only the i variants with the smallest single variant *p*-value. Below each point, the minor allele count of the added variant is given.

For an unbiased evaluation of the imputation quality, we used 181 WES samples not included in the reference panel and compared imputed dosages and hard calls of sequencing-derived genotypes at sites with MAC ≥ 2 in both callsets (*n* = 188,397). The mean and median squared correlation of imputed dosage and sequencing hard calls (R2) was 0.913 and 0.978, respectively (Figure 2a). Rare variants with a MAC between 2 and 10 in the reference panel achieved a mean and median R2 of 0.96 and 1.00, respectively. The overall genotype hard call concordance was 0.989 with a concordance of 0.995, 0.963, and 0.975 for homozygous reference, heterozygous, and homozygous alternative calls, respectively (Figure 2b). To compute the true positive rate (TPR) and true negative rate (TNR), heterozygous and homozygous alternative genotypes (i.e., carriers of variant) were defined as “positive” and homozygous reference genotypes (i.e., non-carrier) defined as “negative”. Using these definitions, the TPR (carriers correctly imputed as carriers) was 0.967, the TNR (non-carriers correctly imputed as non-carriers) was 0.995, the false negative rate (carriers incorrectly imputed as non-carriers) was 0.019, and the false positive rate (non-carriers incorrectly imputed as carriers) was 0.005.

### 2.2. Single Variant Associations

We identified 85 locus-trait associations at 54 unique loci in 40 unique traits significant at a *p* < 5.5 × 10^−9^ threshold (Methods) in the WES combined set (*n* = 5505; Table S4). Of the 85 associations, 39 associations at 29 loci in 28 traits remained significant at a 5.5 × 10^−9^ threshold in the WES combined dataset after adjusting for known common variant signals (Table 1, Figure 2c and Figure S4). Of the 39 associations, 13 (6 rare) were near known variants (±1 Mb of index variant), but conditionally independent (secondary signals). The remaining 26 associations (10 rare) did not have known variants nearby (novel signals, Figure S5). Of the 29 unique index variants, 9 were directly genotyped and 20 imputed with high imputation quality (median rsq = 0.92, range 0.71–1.0). The index variants explained 0.03% to 8.2% (median = 0.85%) of the variance in the respective traits. Twenty of the twenty-nine unique variants were enriched in our data compared to the gnomAD v2.1.1. European (non-Finnish) population.

**Figure 3:**
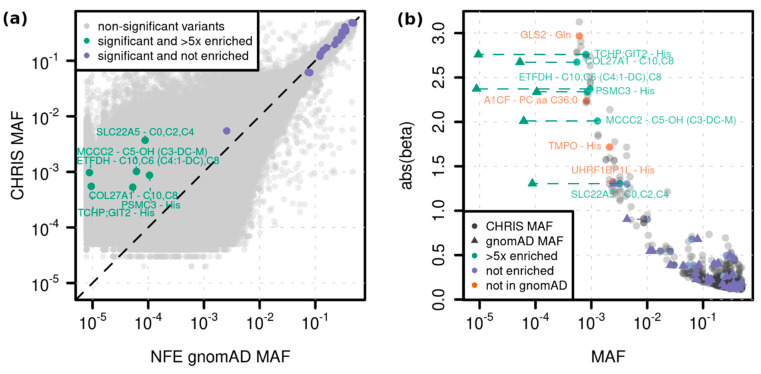
Variant enrichment in CHRIS compared to gnomAD. (**a**) Minor allele frequency (MAF) of matching variants in CHRIS and gnomAD for variants with a minor allele count greater zero in both cohorts are plotted in grey. For gnomAD the non-Finnish European (NFE) MAF is plotted using both exome and genome sequencing data. Variants with conditionally significant annotations in CHRIS are plotted in color, with variants enriched at least five-fold in CHRIS colored in green and labeled with gene—trait(s). (**b**) CHRIS MAF versus the absolute value of beta is plotted as grey points for all significant single variants. The conditionally significant associations listed in Table 1 are colored. For variants that existed in gnomAD, the gnomAD NFE MAF is plotted as triangles in green (>5 times enriched) or purple (<5 times or not enriched) and connected for visibility. For variants that did not exist in gnomAD or had an allele count of zero, only the CHRIS MAF is plotted in orange. Associations from Table 1 with an absolute beta value greater than one are annotated with gene—trait(s).

Including four variants that were not detected in gnomAD, 10 variants in 15 associations were more than five-fold enriched in the CHRIS population, which demonstrates the value of this cohort and its power to identify associations for variants rare or absent in other populations (Figure 3). For example, the secondary association at the *GLS2* missense variant p.Ser500Pro (12:56866487:A:G) with glutamine (*p* = 3 × 10^−14^, MAF = 0.001, beta = −2.97) is not present in gnomAD. *GLS2* encodes glutaminase 2, an enzyme that catalyzes the conversion of glutamine to glutamate and ammonia, promoting mitochondrial respiration and ATP generation. Another example is the novel association at the *UHRF1BP1L*/*ACTR6* splice region variant 12:100492127:T:C with histidine (*p* = 2.6 × 10^−9^, MAF = 0.02, beta = 1.32). This variant is in high LD (r^2^ = 0.98) with the *ACTR6* missense variant p.Arg36His (rs772372420, *p* = 4 × 10^−8^, MAF = 0.002, beta = 1.2). The association was significant only before conditional analysis, but was predicted as deleterious (SIFT = 0, PolyPhen = 0.997, CADD = 31). Little is known about the role of *ACTR6*, and therefore, this gene might be a novel causal gene for histidine regulation.

Of the 39 conditionally independent single variant associations, three are in genes related to recessive inherited metabolic disorders. These are the associations *MCCC2*—hydroxyvalerylcarnitine (3-methylcrotonylglycinuria), *ETFDH*—hexanoylcarnitine (glutaric acidemia type II) and *SARDH*—sarcosine (sarcosinemia). Of the three variants, the *ETFDH* stop gain variant p.Trp286Ter (rs1235904433, *p* = 8 × 10^−15^, beta = 2.4, MAF = 0.001, rsq = 0.78, CHRIS enrichment = 110x) and the *MCCC2* stop lost variant p.Ter564GlnextTer3 (rs751970792, *p* = 2 × 10^−12^, beta = 2.0, MAF = 0.001, rsq = 0.88, CHRIS enrichment = 17x) have been reported in individuals with the respective diseases glutaric acidemia type II and 3-methylcrotonylglycinuria, compatible with a recessive disease mode [12,13,14,15]. Glutaric acidemia type II is commonly identified in newborn screenings by the elevation of two or more acylcarnitines, such as hexanoylcarnitine [12]. Additionally, for 3-methylcrotonylglycinuria, a characteristic clinical phenotype is the presence of hydroxyvalerylcarnitine in blood and urine [14]. For both rs1235904433 and rs751970792, we observed only heterozygous individuals in our cohort, which, despite elevated metabolite levels, appear phenotypically healthy. Both variants represent examples of rare, novel associations enriched more than five-fold in CHRIS, which, even though they have been associated with the disease, they have not been reported as associated the metabolite in a GWAS previously. The third variant rs10993780 is a common intron variant in *SARDH* with 136 homozygous healthy carriers in our cohort, which indicates that it is not causal for sarcosinemia.

To facilitate the discussion of the identified associations, we assigned them to one of three categories based on previous biological knowledge of the relationship between genes and associated metabolites (Table 2): (1) variants in enzymes acting directly on the associated metabolite, (2) variants in other enzymes in the metabolic pathway, or (3) variants in genes with no established link to the metabolite or its direct metabolic pathway.

Twelve associated variants were in enzymes that act directly on the metabolite. One example is the novel association of *SLC22A1* inframe insertion variant p.Met420dup with serotonin (rs72552763, *p* = 1 × 10^−11^, MAF = 0.16, beta = 0.19). *SLC22A1* encodes the organic cation transporter protein (OCT1), which has selectivity for serotonin among other endogenous molecules and drugs. Therefore, a variant on this gene can directly affect the plasma concentration of serotonin [16].

Another 12 associations involved enzymes in the metabolic pathway of the metabolite (level 2). One example is the association of the CHRIS-enriched *ETFDH* stop gain variant p.Trp286Ter with octanoylcarnitine (rs1235904433, *p* = 7 × 10^−18^, beta = 2.5, MAF = 0.001, rsq = 0.78, CHRIS enrichment = 110x). *ETFDH* encodes for a protein involved in the electron-transfer chain (ETC) in mitochondria, while octanoylcarnitine is a metabolite involved in the fatty acid β-oxidation (FAO). Both ETC and FAO are mitochondrial metabolic pathways that are required for energy production in the mitochondria. Variants in *ETFDH* might also affect other mitochondrial metabolic pathways, such as FAO, and therefore the blood level of some acylcarnitines, even though the mechanism leading to an altered level of octanoylcarnitine is not known [17].

For the remaining 15 associations, no clear link between the putative causal gene and the metabolite could be established (level 3). This latter class represents associations that might offer novel insights into the genetic determinants of metabolite levels and merit follow-up. One example of a putatively novel gene association is the association of the common *PPID* missense variant p.Leu302Ile (rs9410, MAF = 0.3) with decenoylcarnitine (*p* = 3 × 10^−11^, beta = −0.14) and dodecanoylcarnitine (*p* = 5 × 10^−14^, beta = −0.17). Even though the index variant is in LD (r^2^ = 0.8) with a significant variant in *ETFDH*, which has been associated with various carnitines previously [18,19], it shows colocalization not only with increased *ETFDH* expression (PP = 0.99), but also with decreased *PPID* expression (PP = 0.69).

### 2.3. Gene-Level Associations

The overwhelming majority of exome variants are rare, and thus we have limited power to detect associations for single variants. To increase power, we performed aggregate tests of putatively functional variants within each gene, combining rare variants of “high” and “moderate” impact as defined by Ensembl (“high-moderate impact” mask) or rare loss of function variants (“loss of function” mask, Methods). Nineteen gene-trait associations were significant at a 3.6 × 10^−8^ threshold (Methods) in the WES combined dataset, 18 with the “high-moderate impact” mask and one with the “loss of function” mask. Sixteen of these associations were driven by a single variant (Table S5, Figure S6), one association (carnitine—*SLC22A5*, *p* = 2.9 × 10^−10^) reached significance only after including the two best variants (rs139203363 and rs202088921), and two gene-trait associations (tryptophan—*TDO2*, *p* = 8.9 × 10^−9^ and sphingomyelin C18:0—*CERS4*, *p* = 6.1 × 10^−10^) reached significance only after including the three best variants (*TDO2*: rs151132024, rs183821149, 4:156837056:T:A; *CERS4*: 19:8321859:C:G, rs1478814187, rs150540280) (Table 3, Figure 2d–f and Figure S7). Neither of the two latter associations were identified in the single variant analysis, which demonstrates the value of gene level association testing.

All three of these gene-based associations involve enzymes that act directly on the associated metabolite. Approximately 95% of tryptophan (Trp) is catabolized via the kynurenine pathway [21] by the tryptophan 2,3-dioxygenase (TDO2), an enzyme encoded by the *TDO2* gene [22]. Therefore, impaired activity of the TDO2 enzyme results in Trp accumulation. The first confirmed case of hypertryptophanemia due to TDO2 deficiency was caused by heterozygosity on two rare variants of *TDO2* gene [21]. The variant carriers from CHRIS have elevated Trp levels (mean = 70.65 μmol/L, max = 96.66 μmol/L; non-carriers mean = 61.88 μmol/L, max = 132.78 μmol/L) and lower blood cortisol levels (two-sided wilcoxon test, *p* = 0.016). Cortisol is known to act as an activator of *TDO2*. Consequently, lower cortisol levels increase Trp availability. Tryptophan levels have been previously associated with behavioral disorders [23,24] such as anxiety [25]. We have found that the anxiety trait score, as calculated from STAI anxiety questionnaire [26] was higher in the carriers than in the non-carriers (one-sided Wilcoxon test, *p* = 0.041), which further supports that tryptophan levels can indeed be correlated with behavioral disorders.

### 2.4. Colocalization Analysis

**Figure 4:**
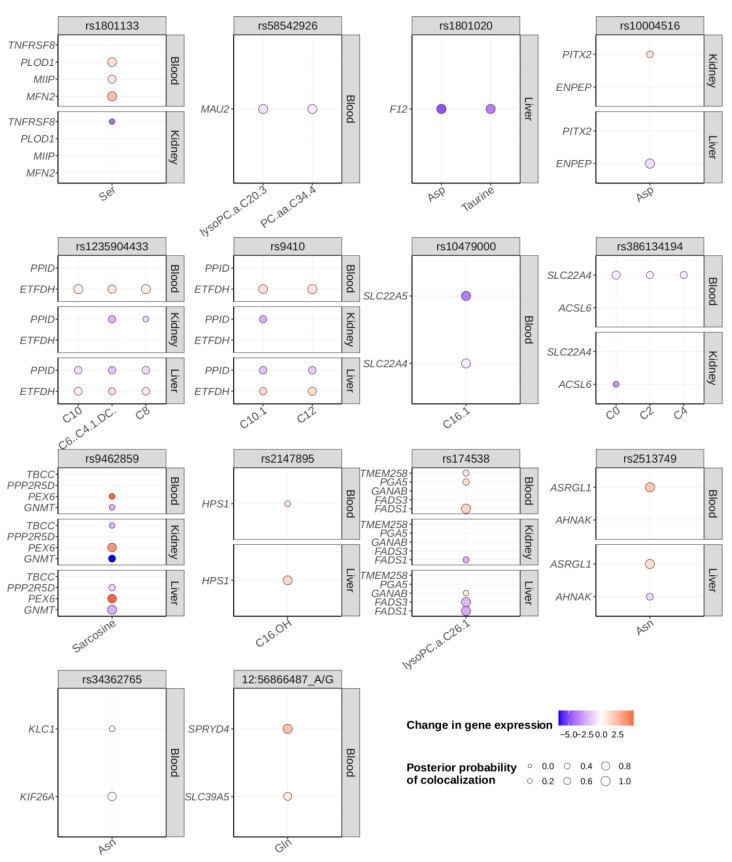
Results of the colocalization analysis. For each index variant with at least one gene colocalized at a posterior probability (PP) ≥ 0.8 in at least one of the three tissues whole blood (Blood), kidney cortex (Kidney), or liver (Liver), colocalization data of all protein coding genes and traits with PP ≥ 0.3 are displayed. The colors represent the change in gene expression relative to an increase in the colocalized metabolite level.

The colocalization analysis revealed 27 locus-trait associations at 14 index variants colocalized with expression quantitative trait loci (eQTL) with a posterior probability (PP) ≥ 0.8 (Figure 4, Table S6). In 17 of the 27 metabolite eQTL colocalizations, the index variant of the metabolite association was either located in or was in high LD with a significant variant in the same gene as the gene whose expression it was colocalized with.

These data allow us to further speculate on the functional genes of the identified associations. For example, a 5′ UTR variant in *TMEM258*, rs174538, was associated with decreased lysophosphatidylcholine acyl C26:1 levels in our data, yet for the corresponding transcript, no link to the metabolite could be found. However, the index variant is in LD with significant variants in *FADS1* (r^2^ = 0.84) and *FASD2* (r^2^ = 0.84) and colocalized with *FADS1* and *FADS3* transcript levels at a PP = 0.97 (Figure 4). Since the fatty acid desaturase enzymes regulate unsaturation of fatty acids through the introduction of double bonds into the fatty acyl chain, a gene of the FADS family is likely the causal gene at this locus. Another example is rs10479000, located in an intron of both *P4HA2* and *PDLIM4*, which is associated with hexadecenoylcarnitine, but none of the eQTLs for either gene colocalizes with the metabolite. However, the index variant is in LD with an upstream variant of *SLC22A5* (r^2^ = 0.82) and colocalized with eQTLs for both *SLC22A5* and *SLC22A4* at a PP = 0.96. Since *SCL22A5* is an organic cation transporter with high affinity for carnitine and we have identified an additional association of the variant rs386134194 in this gene with butyrylcarnitine and carnitine, it is likely that *SLC22A5* is the functional gene for the rs10479000 signal as well.

### 2.5. Mendelian Randomization

We then used the genetic associations from each metabolite as instrumental variables in Mendelian randomization analyses to test whether metabolite levels could be causally linked to health-related outcomes using the UK Biobank. We identified 63 significant metabolite-outcome associations. The overwhelming majority, 62 of 63, exhibited some evidence of pleiotropy, which is a violation of the assumptions of Mendelian randomization, and so must be interpreted with caution with respect to causal inference. One association, putrescine to waist circumference (*p* = 1.8 × 10^−12^), though, did not exhibit evidence of pleiotropy and is consistent with previous studies. Increased putrescine has been associated with obesity in children [27] and with type 2 diabetes [28]. Seven metabolites had at least one significant outcome association (carnitine = 30, phosphatidylcholine diacyl C34:4 = 12, propionylcarnitine = 9, lysoPhosphatidylcholine acyl C20:3 = 6, putrescine = 4, hexanoylcarnitine (fumarylcarnitine) = 1, serine = 1). Forty-eight outcomes were associated with at least one metabolite (Table S8).

## 3. Discussion

We performed an exome-wide association study on 175 metabolic traits in 5505 individuals from a North Italian Alpine valley (CHRIS study). Since only a subset of individuals (*n* = 3294) was whole-exome sequenced, we used these sequenced individuals and exonic variants as a reference panel for within-cohort imputation for the remaining 2211 individuals (Figure 1). This strategy generated a set of high-quality genotypes with a mean squared correlation R2 of 0.91 over all tested variants.

The ExWAS in the combined dataset revealed 85 variant-trait associations, including 39 conditionally independent of known common variant signals, and 3 of 19 significant gene-trait associations showed contribution from multiple rare variants (Figure 2). Twelve of the novel thirty-nine associations were located in an enzyme acting directly on the metabolite (level 1), twelve associations were located in an enzyme that was involved in the metabolic pathway of the metabolite (level 2), and for the remaining fifteen associations, no link between the putative gene and the metabolite could be identified (level 3).

Fifteen associations emerged at ten variants which were enriched more than five-fold compared to the non-Finnish population in gnomAD (Figure 3). We identified three associations related to inherited metabolic disorders. Of these, the *ETFDH* stop gain variant p.Trp286Ter (rs1235904433, *p* = 8 × 10^−15^ with hexanoylcarnitine) and the *MCCC2* stop lost variant p.Ter564GlnextTer3 (rs751970792, *p* = 2 × 10^−12^ with hydroxyvalerylcarnitine) have been linked to the respective diseases glutaric acidemia type II and 3-methylcrotonylglycinuria but have not been identified in a GWAS previously.

Colocalization analysis with GTEx gene expression data analysis revealed 27 locus-trait associations colocalized with eQTLs at a posterior probability ≥ 0.8. Mendelian randomization analysis with health-related outcomes in the UK Biobank resulted in 63 significant metabolite-outcome associations, 62 with some evidence of pleiotropy and one without any evidence of pleiotropy (putrescine—waist circumference *p* = 1.8 × 10^−12^).

Even though previous metabolite GWAS have benefited from a larger sample size or more metabolites tested, our study queries the entire coding space, which, due to the high cost of sequencing is infeasible for many mGWAS studies, who often use imputed genotype data [5,6,7,8]. Despite the smaller size of our panel compared to others, the Biocrates kit used here measures some metabolites missed by other common platforms, such as Metabolon [5,6,8,29,30] or Nightingale [6,11]. For example, Nightingale does not measure the amino acids tryptophan, arginine, or aspartate, and neither Metabolon nor Nightingale measures the biogenic amines histamine, spermine, or putrescine, or the acylcarnitines decadienylcarnitine or dodecanedioylcarnitine.

There are potential limitations to our study. First, the CHRIS cohort only includes individuals of European ancestry; therefore, our results might not be generalizable to non-European ancestries. Second, we favor a joint analysis approach over a discovery and replication setting since it has been shown that joint analysis is more powerful than replication [31]. Nonetheless, we report also the results for the whole-exome sequenced data set and the imputed data set separately. We acknowledge that external replication could provide additional evidence. Finally, we use the EBI GWAS catalog in addition to a literature search to identify known associated variants to use in the conditional analysis. However, new associations are identified at a high pace and very recently reported associations might not be included in our conditional analysis.

The unique characteristics of the CHRIS study represent a clear strength of this work. The participants were recruited from an Alpine valley in Northern Italy, where the individuals share similar heritage, lifestyle, and diet habits that are influenced by tradition and rural culture. While the region was not geographically isolated in the past hundreds of years, gene-flow was certainly limited in contrast to more urban areas, potentially leading to the collection of enriched variants we identified in this study.

Several studies have highlighted the value and challenges of WES imputation [9,32,33]. In our study, we demonstrate that this approach is a cost-efficient strategy to infer coding variation in individuals not sequenced. Achieving high quality imputation is especially challenging for rare variants. Using a true validation set of individuals that have been sequenced as well as imputed, we demonstrate high imputation accuracy at very rare variants (mean R2 = 0.96 for *n* = 6263 variants with MAC 2–10 in the reference panel). In fact, the imputation accuracy at rare variants even supersedes the accuracy at more common variants (Figure 2a,b), which might be due to the high relatedness of the individuals in the CHRIS cohort, as haplotype matching might be more accurate. Of the 39 significant associations, only 20 would have been detected using only the WES subset of 3294 individuals, which demonstrates the increase in power achieved by the exome imputation. Apart from the ability to identify rare variants, (imputed) whole exome sequencing data by design focuses on the protein coding part of the genome, which facilitates the interpretation of results in contrast to the non-coding genome.

To sum up, we show the value of smaller, well-characterized cohorts in the age of large population biobanks like the UK Biobank through the identification of 15 associations at 10 variants that are enriched in CHRIS. We almost doubled our sample size and therefore our power to detect associations in the coding space using a within-cohort imputation strategy. Our results extend the knowledge about genetic mechanisms controlling human metabolism with the potential for identifying novel targets and impacting human health.

## 4. Materials and Methods

An overview of the study workflow is given in Figure 1.

### 4.1. CHRIS Population Study

The Cooperative Health Research in South Tyrol (CHRIS) study [10] is a single-site, prospective, population-based study with 13,389 participants recruited from the Vinschgau/Val Venosta valley in South Tyrol, Italy, between 2011 and 2018. The study was initiated with the goal of investigating the relationship of genetic, metabolomic, environmental and lifestyle factors with common chronic conditions, emphasizing neurological and cardiovascular diseases. Participants underwent tremor assessment, blood drawing, urine collection, anthropometric measurements, a 20 min electrocardiographic (ECG) analysis, and blood pressure measurement. Additionally, each participant completed an interview with questions to screen for cardiovascular, endocrine, metabolic, genitourinary, nervous, behavioral and cognitive system conditions, and to semi-quantitatively assess nutrient intake, physical activity, and life-course smoking.

### 4.2. Genotyping

DNA samples were genotyped using the Illumina HumanOmniExpressExome and Illumina Omni2.5Exome array. Illumina GenomeStudio v2010.3 with default settings was used to call genotypes on GRCh37. Variants with GenTrain score < 0.6, cluster separation score < 0.4, or call rate < 80% were considered technical failures and discarded. Only variants present on both arrays were forwarded to our standard quality control pipeline. Samples with a call rate < 98%, monomorphic variants or variants with Hardy-Weinberg equilibrium *p* < 10^−6^ were removed. After quality control, 612,000 variants and 10,770 samples were retained.

### 4.3. Whole Exome Sequencing

Whole exome sequencing (WES) of 3840 CHRIS participants was performed using the xGen^®^ Exome Research Panel v1.0 at the McDonnell Genome Institute at Washington University. The generated exome sequencing data were processed using the Genome Analysis Toolkit (GATK) v3.7 best practices pipeline [34,35,36] in conjunction with additional quality control measures. Specifically, reads were aligned with BWA version 0.7.15 [37] to GRCh37, and duplicates annotated with SAMBLASTER [38]. Base quality scores were recalibrated with GATK and quality control statistics generated with Qualimap v2.2.1 [39] and QPLOT ver: 20,130,619 [40]. Samples were excluded if they did not have more than or equal to 20X coverage at a minimum of 80% of target sites (176 samples). VerifyBamID version 1.1.2 was used to detect the contamination of samples with foreign DNA, and to determine sample swaps by comparison with the genotype array data [41]. Samples with greater than 3% contamination were removed (*n* = 79, FREEMIX > 3%). Forty-two cases of sample swapping were detected, six of which could be reassigned. Using QPLOT, 127 samples were identified as having abnormal q20 bases vs. cycle plots and were removed. After quality control, 3422 samples were retained, with a mean target coverage of 68.4X. For a subset of 3294 samples, metabolite data were available and were used subsequently. All 3294 sequenced samples were also genotyped.

Variant calling was performed on the exonic regions defined in the xGen^®^ Exome Research Panel v1.0 bed file with an interval padding of 500 bp. Per-sample variant calling was performed with the GATK HaplotypeCaller, followed by joint-call genotyping across all samples using GATK’s GenotypeGVCFs. Variant quality score recalibration was performed, and variants were filtered based on a tranche sensitivity threshold of 0.99 for both SNVs and indels. Post GATK, the software vt version 0.5772 [42] was used to decompose multiallelic sites and to normalize variants. Further variant annotation was performed with the Ensembl Variant Effect Predictor (VEP) version 99 [43] with the Loss-Of-Function Transcript Effect Estimator (LOFTEE) plugin [44] to identify high confidence loss of function variants. The mean concordance between hard call genotypes called from the WES data and the array genotype data (number of matching genotypes/total number of genotypes) was 0.998 for the approximately 25,000 overlapping variants. After quality control, 1,121,060 variants were retained.

### 4.4. Genotype Imputation

*Custom Reference Panel.* Imputation of a population specific whole-exome sequencing reference panel into 10,770 genotyped CHRIS individuals was performed. To create the reference panel, all WES variants of the 3294 sequenced individuals were combined with the genotypes for the same set of individuals. If a variant was present both in the genotyping and the sequencing data, genotyping data were used. Variants were phased with SHAPEIT2 v2.r837, using the duoHMM method (--duohmm -W 5) with 800 states and 30 rounds [45]. Genotypes of all 10,770 samples were phased with SHAPEIT2 using the same parameters as for the reference panel. Imputation was performed with mimimac3 version 2.0.1, using 800 states and 20 rounds [46]. Variants with estimated imputation quality rsq < 0.3 were removed. Validation of imputation quality was performed on 181 imputed samples which underwent WES belatedly in the same fashion as the 3294 samples described above and were not included in the reference panel nor in the ExWAS described in this paper. For the validation, genotyped variants were removed.

*Standard Reference Panel.* To enlarge the set of variants available for conditional analysis, imputation of the TOPMed reference panel into the 10,770 genotyped CHRIS samples was performed with the Michigan imputation server [47,48]. Variants with rsq < 0.3 were removed.

### 4.5. Metabolomics Data

Measurement, data normalization and quality assessment of the targeted metabolomics data are described in [49]. In brief, the AbsoluteIDQ p180 kit (Biocrates Life Sciences AG, Innsbruck, Austria) was used to determine absolute concentrations for 188 metabolites in serum samples from participants of the CHRIS study. To remove batch effects, the data were normalized using a combination of 3 different quality control (QC) samples, included on each plate. Quality assessment was based on the number of missing values, coefficient of variation across QC samples and visual inspection of signal distributions. Thirteen of the one hundred and eighty-eight metabolites were removed because of poor quality.

In order to obtain a homogenous set of individuals for the analysis, pregnant and possibly pregnant women as well as individuals of non-European descent (both self-reported) were excluded from the analysis, resulting in a data set of 175 quantified metabolites in 5505 individuals. All these 5505 individuals were genotyped and 3295, in addition, exome sequenced.

Concentrations for each metabolite were further adjusted for age, sex, fasting status (categories: did fast (93.3%), did not fast (6.6%), not available (0.1%)), and the first ten principal components using linear regression, followed by rank inverse normal transformation of the model’s residuals. All genetic analyses were performed using these transformed residuals. Additionally, the single variant ExWAS was repeated correcting also for body mass index (BMI) and genotyping batch prior to the rank inverse normal transformation. Since the *p*-values were highly correlated (0.996 and 0.987, Figure S1), results are reported here for the analysis without correcting for BMI or genotyping batch. The metabolites analyzed in this study are displayed in Table S1.

To choose a significance threshold for single variant and gene tests, principal component analysis (PCA) was performed on the 175 transformed traits. Missing trait values were imputed with the imputePCA function of the missMDA R package. Principal components (PCs) were computed with the PCA function of the FactoMineR package and the prcomp function of the stats package. The first 91 PCs explained 95% of the variability; therefore, the *p*-value threshold was set to 0.05/(100,000 × 91) = 5.5 × 10^−9^ for single variant tests [50] and to 0.05/(15,496 × 91) = 3.5 × 10^−8^ for gene tests, as 15,496 was the maximum number of genes tested in either mask. Trait correlation is displayed in Figure S2.

The Biocrates platform predefines 29 and 36 biologically relevant sums and ratios, respectively, from the basic measured metabolites (Tables S2 and S3). Since these derived traits are highly informative for biological interpretation but increase the multiple testing burden, the ExWAS was only performed on the 175 basic metabolites. Subsequently, association tests were performed for the sums and ratios only on the index variants of the significant associations. Rank inverse normal transformations were performed on the ratios and sums were as described for the basic metabolites.

### 4.6. Definition of Datasets

For the analysis, three datasets were defined (autosomes only). Association analysis was performed on variants with minor allele count (MAC) ≥ 4.

1. Whole exome sequencing (WES): All individuals with whole-exome sequencing and measured metabolite data (3294 individuals and 554,589 variants).
2. Imputed only (WES imputed): All individuals with genotype data (and thereby imputed) that were not in the imputation reference panel with measured metabolite data, restricting to imputed variants only (2211 individuals 374,349 variants).
3. Whole-exome sequencing combined with imputed (WES combined): All individuals with whole-exome sequencing data, genotype, and imputation data, and with measured metabolite data, combining sequenced, genotyped, and imputed variants (5505 individuals and 624,751 variants).

The final evaluation of results was performed on the WES combined set, but summary statistics for the WES and WES imputed set are provided in Table S4.

### 4.7. Known Genetic Associations and Conditional Analysis

Literature mining was performed to identify previously reported GWAS associations between metabolites and genetic variants. The identified trait descriptions were manually mapped to Biocrates metabolite IDs based on the provided trait names, common identifiers, such as HMDB IDs, or metabolite descriptions. Additionally, the EBI GWAS catalog r2020-11-20 was mined for known associations (Appendix A).

At the time of writing this manuscript, 2197 previously reported genome-wide significant associations were identified in 115 traits, corresponding to 1746 unique variants. For the remaining traits no reported associations could be found. Genomic coordinates for these associations were obtained in GRCh37 and GRCh38. Variants were extracted from WES imputed or TOPMed imputed datasets with preference given to WES imputed over TOPMed in case the variant was present in both datasets. In total, 1678 (96%) variants were present in at least one dataset.

Novel associations were categorized into 3 levels reflecting their biological relevance. Level 1—The gene is encoding an enzyme that acts directly on the metabolite. Level 2—The gene encodes an enzyme that acts on a metabolite present in the same metabolic pathway as the associated metabolite. Level 3—No relationship between the gene and the metabolite could be found.

### 4.8. Single Variant Association Tests

Single variant association tests on all 175 traits were performed on the rank inverse normal transformed metabolite concentrations using the q.emmax test of the EPACTS version 3.2.6 software on all variants with a minor allele count greater three. Tests were performed on the WES, the WES imputed and the WES combined set separately, using genotype hard calls for WES and dosage values for the latter two datasets.

The significance threshold was set to 0.05/(100,000 × 91) = 5.5 × 10^−9^ as described above. For 115 of the 175 traits, variants meeting the traditional genome-wide significance threshold of *p* ≤ 5 × 10^−8^ have been reported previously. For these traits, the association tests were repeated in all three datasets conditioning on all known variants for each trait.

Subsequently, single-variant tests were performed on the 29 predefined sums and 36 predefined ratios only for the index variants that reached conditional significance.

To identify the independent loci for each metabolite, we applied LD-based clumping with swiss version 1.1.1 [51] on the unconditional ExWAS in the WES combined set using the phased WES combined data as LD source with a clump *p*-value threshold of 5.5 × 10^−9^ and an LD clumping threshold of 0.8. Since LD-based clumping is not always successful, distance-based clumping with swiss was performed subsequently using a 500 kb window and a clump *p*-value threshold of 5.5 × 10^−9^. This procedure resulted in 112 loci in 40 traits. On these 112 loci, the following filtering steps were performed: (i) remove variants that could not be tested in the WES set due to MAC < 4 (*n* = 23), (ii) remove variants that have a higher alternative allele count in the WES imputed set than in the WES set (*n* = 4), (iii) remove variants where the direction of effect was different in the WES imputed and the WES set (*n* = 0). This filtering procedure resulted in 85 loci in 40 traits that were significant at a 5.5 × 10^−9^ threshold (Table S4). After conditional analysis, 39 locus-trait associations remained significant at a 5.5 × 10^−9^ threshold in 28 unique traits and 29 unique variants (Table 1).

To determine the causal gene for the detected associations, we used the variant effect predictor (VEP) version 100 to annotate the index variants. If only one gene overlapped the variant, we reported this gene and the most severe consequence [52]. If multiple genes overlapped the variant, we reported only the gene with the most severe consequence. We further extracted all significant variants (*p* ≤ 5.5 × 10^−9^) in high LD (r2 > 0.8) with the index variant and reported the corresponding gene (Table 1 and Table 2).

### 4.9. Gene Level Association Tests

To increase power to identify rare-variant signals, we performed gene-level association tests using the R-package SKAT [53]. All variants in the three datasets were annotated with Ensembl v99 data, using a local installation of VEP with the plugin LOFTEE to annotate loss of function variants. Variants were grouped across genes using two different masks. For the “loss of function” mask, variants were included for a gene if the variant (i) was annotated as high confidence loss-of-function variant (as determined by the VEP LOFTEE plugin) in at least one protein-coding gene transcript and (ii) had a minor allele frequency (MAF) of at most 0.01 in the analyzed individuals. For the second mask “high-moderate impact”, variants were included for a gene if the variant (i) had an annotated consequence that was either “high” (transcript ablation, splice acceptor variant, splice donor variant, stop gained, frameshift variant, stop lost, start lost, transcript amplification) or “moderate” (in-frame insertion, in-frame deletion, missense variant, protein altering variant) as defined by Ensembl [52] in at least one protein-coding gene transcript and (ii) had a MAF ≤ 0.01 in the analyzed individuals. Genes were tested for association in either mask, if at least three variants fulfilled these criteria. Specifically, for each trait, the SKAT_NULL_emmaX function from the SKAT R-package was used to build the model, including the kinship matrix. For each gene, the SKAT function with method “skato” was invoked.

To exclude spurious associations that are driven by one highly associated variant, the contribution of the individual variants to the gene level *p*-value was determined for all gene-trait associations that reached significance in the combined dataset. For each dataset, the *p*-values of the constituting variants were extracted from the single variant association tests. Variants were ordered by increasing *p*-value, and the skato test was performed stepwise, starting with only the best variant by *p*-value, subsequently adding one variant and repeating the test, finally performing the test on all constituting variants. A gene-trait association was only considered genuine, if significance in the WES combined set was reached after adding at least two variants. Furthermore, the *p*-value in the combined set was required to be smaller than the *p*-value in the WES set.

### 4.10. Colocalization Analysis

To identify shared causal variants between the tested metabolites and gene expression, colocalization analysis of the conditionally significant single variant associations with expression quantitative trait loci (eQTL) associations was performed using GTEx data version 8 (EUR) for the kidney, whole blood, and liver tissue. All genes within a ±1 Mb window of the ExWAS index SNPs were tested for colocalization using the coloc.fast function of the gtx R-package version 2.1.6 [54], which is an implementation of the colocalization method described here [55]. For each colocalization analysis, all variants within ±100 kb of the index variant were included using the default prior probability of 1 × 10^−5^. A posterior probability (PP) ≥ 0.8 was considered as an indicator that the same locus is causal for both the metabolite and the eQTL association.

### 4.11. Mendelian Randomization

Causal effects of differences on metabolite concentrations on 172 functional related outcomes (i.e., blood, urine and health-related traits) were tested by a two-sample Mendelian randomization (MR) approach [56], using summary genetic data obtained from two independent homogeneous populations: this ExWAS study (*n* = 5505 all with European ancestry) for metabolites and the Pan-UKBiobank study (*n*~500,000 with a great majority of European ancestry [57]) (Table S7).

For each of the 175 metabolites, genetic instruments were selected that were genome-wide significant (*p* ≤ 5 × 10^−8^), had sufficient strength (F-statistic > 10), and were pairwise independent (LD clumping with r^2^ < 0.01). The F-statistic was estimated by the ratio between the squared estimate of the instrument-metabolite association and its squared standard error [58]. LD clumping was performed with swiss [51]. Genetic instruments not present in Pan-UKBiobank were discarded since no proxies could be identified for these mostly rare variants.

After instrument selection, genetic data were harmonized [56], that is, first, negative genetic effect estimates on metabolites were flipped, with its effect allele and the corresponding frequency. Furthermore, an alignment was created by effect allele and frequency of the outcome. To guarantee the homogeneity between the two genetic datasets, genetic effect estimates on the outcome were also flipped when there was no correspondence with the allele frequency of the metabolite.

No MR analyses were performed on metabolites with fewer than four instruments since the presence of pleiotropy could not be investigated with statistical tools. Hence, the pleiotropy was evaluated for 11 metabolites using the I2 index and the Cochran Q test [59]. A nominal significance threshold was used. The following MR methods were applied for 1826 metabolite-outcome causal hypotheses: the inverse variance weighted random effects (IVW-RE) [60], MR-Egger (MRE) [61], weighted median (WMedian) [62], and weighted mode-based (WMode) estimators [63]. Analyses were performed using the R software (version 4.1.1) and MendelianRandomization R package (version 0.5.1).

We implemented both IVW-RE and MR-Egger using MM-estimation and penalization of extreme Wald estimates, an approach robust to outliers [64]. Standard errors of WMedian and WMode estimates were estimated using a bootstrap procedure with 10,000 iterations (default setting in MendelianRandomization package). Moreover, when the algorithm of one of the MR methods did not converge, the MR result was removed.

The significance threshold was set to α = 0.05/1826 = 2.74 × 10^−5^. For metabolite-outcome pairs with no evidence of pleiotropy, a causal association was called significant if the *p*-value of IVW-RE test was below α. When there was statistical evidence of pleiotropy, a causal association was called significant if the estimates of the MRE, WME, and WMO tests were direction consistent, and all *p*-values were smaller α. A rank of statistical evidence of causality was defined by identifying the number of significant MR estimates. The overall MR procedure is described in Figure S3.

## Figures and Tables

**Table 1:** Locus-trait associations conditionally significant at a 5.5 × 10^−9^ threshold in the WES combined dataset.

Trait Code	Trait Name	Gene (LD Mapped Gene)	Variant (Rsid)	Effect	*p*-Value (Conditioned)	Beta (SE)	MAF
Ser	Serine	*MTHFR*	1:11856378_G/A (rs1801133)	missense	3.4 × 10^−8^ (4.0 × 10^−9^)	−0.11 (0.02)	0.405
Asp	Aspartate	*ENPEP*	4:111398208_A/G (rs10004516)	missense	1.2 × 10^−7^ (1.8 × 10^−11^)	0.16 (0.03)	0.127
C10	Decanoylcarnitine	*ETFDH*	4:159618737_G/A (rs1235904433)	stop gained	1.1 × 10^−12^ (2.5 × 10^−16^)	2.37 (0.33)	0.001
C6 (C4:1-DC)	Hexanoylcarnitine (Fumarylcarnitine)	*ETFDH*	4:159618737_G/A (rs1235904433)	stop gained	2.1 × 10^−13^ (8.0 × 10^−15^)	2.44 (0.33)	0.001
C8	Octanoylcarnitine	*ETFDH*	4:159618737_G/A (rs1235904433)	stop gained	3.4 × 10^−14^ (6.5 × 10^−18^)	2.51 (0.33)	0.001
C10:1	Decenoylcarnitine	*PPID* (*ETFDH*)	4:159631991_G/T (rs9410)	missense	3.8 × 10^−11^ (2.7 × 10^−11^)	−0.14 (0.02)	0.296
C12	Dodecanoylcarnitine	*PPID* (*ETFDH*)	4:159631991_G/T (rs9410)	missense	4.8 × 10^−14^ (5.2 × 10^−14^)	−0.17 (0.02)	0.298
C5-OH (C3-DC-M)	Hydroxyvalerylcarnitine (Methylmalonylcarnitine)	*MCCC2*	5:70952685_T/C (rs751970792)	stop lost	1.9 × 10^−12^ (1.9 × 10^−12^)	2.01 (0.28)	0.001
C16:1	Hexadecenoylcarnitine	*P4HA2; PDLIM4* (*SLC22A5*)	5:131607402_T/C (rs10479000)	intron; intron	2.3 × 10^−10^ (1.3 × 10^−10^)	−0.13 (0.02)	0.489
C2	Acetylcarnitine	*SLC22A5*	5:131714129_G/A (rs386134194)	synonymous	2.4 × 10^−13^ (3.2 × 10^−13^)	−1.38 (0.19)	0.003
C4	Butyrylcarnitine	*SLC22A5*	5:131714129_G/A (rs386134194)	synonymous	1.4 × 10^−10^ (1.3 × 10^−12^)	−1.22 (0.19)	0.003
C0	Carnitine	*SLC22A5*	5:131714129_G/A (rs386134194)	synonymous	5.6 × 10^−12^ (6.5 × 10^−12^)	−1.3 (0.19)	0.003
Asp	Aspartate	*F12; GRK6*	5:176836532_A/G (rs1801020)	5UTR; intron	2.5 × 10^−9^ (3.5 × 10^−10^)	0.14 (0.02)	0.235
Taurine	Taurine	*F12; GRK6*	5:176836532_A/G (rs1801020)	5UTR; intron	5.9 × 10^−16^ (5.9 × 10^−16^)	0.19 (0.02)	0.235
Sarcosine	Sarcosine	*PEX6* (*GNMT*)	6:42946943_G/A (rs9462859)	5UTR	7.8 × 10^−13^ (7.8 × 10^−13^)	−0.16 (0.02)	0.478
C3	Propionylcarnitine	*SLC22A1*	6:160551204_G/C (rs683369)	missense	7.7 × 10^−12^ (1.2 × 10^−19^)	0.17 (0.03)	0.196
Serotonin	Serotonin	*SLC22A1*	6:160560880_CATG/C (rs72552763)	inframe insertion	1.3 × 10^−11^ (1.2 × 10^−11^)	0.19 (0.03)	0.159
Putrescine	Putrescine	*AOC1*	7:150553605_C/T (rs10156191)	missense	2.3 × 10^−1^ (2.5 × 10^−15^)	0.03 (0.02)	0.236
C10	Decanoylcarnitine	*COL27A1*	9:116931401_C/T (rs145560419)	synonymous	8.8 × 10^−9^ (3.2 × 10^−10^)	−2.68 (0.47)	0.001
C8	Octanoylcarnitine	*COL27A1*	9:116931401_C/T (rs145560419)	synonymous	4.7 × 10^−9^ (4.3 × 10^−10^)	−2.67 (0.46)	0.001
Sarcosine	Sarcosine	*SARDH*	9:136598926_C/G (rs10993780)	intron	5.3 × 10^−44^ (5.3 × 10^−44^)	−0.39 (0.03)	0.171
PC aa C36:0	Phosphatidylcholine diacyl C36:0	*A1CF*	10:52603951_AT/A (-)	intron	4.1 × 10^−9^ (5.4 × 10^−9^)	2.23 (0.38)	0.001
Putrescine	Putrescine	*JMJD1C*	10:65225899_A/AGGCGGC (rs3841602)	upstream	1.7 × 10^−19^ (5.2 × 10^−20^)	0.19 (0.02)	0.477
C16-OH	Hydroxyhexadecanoylcarnitine	*PYROXD2*	10:100148308_T/G (rs2147895)	intron	8.9 × 10^−19^ (8.9 × 10^−19^)	−0.18 (0.02)	0.336
His	Histidine	*PSMC3*	11:47445720_G/A (rs186188306)	synonymous	5.5 × 10^−10^ (4.5 × 10^−9^)	−2.34 (0.38)	0.001
lysoPC a C26:1	lysoPhosphatidylcholine acyl C26:1	*TMEM258* (*MYRF, FADS1, FADS2*)	11:61560081_G/A (rs174538)	5UTR	3.3 × 10^−10^ (3.3 × 10^−10^)	−0.14 (0.02)	0.264
Asn	Asparagine	*ASRGL1*	11:62105391_C/T (rs2513749)	5UTR	1.2 × 10^−15^ (7.8 × 10^−19^)	0.24 (0.03)	0.12
Gln	Glutamine	*GLS2*	12:56866487_A/G (-)	missense	8.2 × 10^−13^ (3.1 × 10^−14^)	−2.97 (0.41)	0.001
His	Histidine	*TMPO*	12:98929093_A/G (rs867372792)	3UTR	8.2 × 10^−15^ (5.6 × 10^13^)	1.72 (0.22)	0.002
His	Histidine	*UHRF1BP1L* (*ACTR6*)	12:100492127_T/C (-)	splice region variant	1.3 × 10^−10^ (2.6 × 10^−9^)	1.32 (0.21)	0.002
Phe	Phenylalanine	*PMCH*	12:102591269_G/T (rs200627654)	intron	8.2 × 10^−17^ (1.4 × 10^−14^)	1.3 (0.16)	0.004
His	Histidine	*TCHP; GIT2*	12:110385016_A/AG (-)	intron; intron	4.2 × 10^−12^ (1.2 × 10^−11^)	2.76 (0.4)	0.001
Asn	Asparagine	*ASPG*	14:104576448_G/A (rs34362765)	intron	8.8 × 10^−105^ (5.8 × 10^−25^)	−0.46 (0.02)	0.358
C10	Decanoylcarnitine	*ABCC1*	16:16139714_T/C (rs35587)	synonymous	4.5 × 10^−8^ (5.0 × 10^−9^)	−0.12 (0.02)	0.326
C12:1	Dodecanoylcarnitine	*ABCC1*	16:16139714_T/C (rs35587)	synonymous	7.2 × 10^−10^ (6.5 × 10^−10^)	−0.13 (0.02)	0.326
C12	Dodecenoylcarnitine	*ABCC1*	16:16139714_T/C (rs35587)	synonymous	1.2 × 10^−9^ (1.2 × 10^−9^)	−0.13 (0.02)	0.326
lysoPC a C20:3	lysoPhosphatidylcholine acyl C20:3	*TM6SF2*	19:19379549_C/T (rs58542926)	missense	9.3 × 10^−9^ (5.0 × 10^−9^)	−0.25 (0.04)	0.054
PC aa C34:4	Phosphatidylcholine diacyl C34:4	*TM6SF2* (*SUGP1*)	19:19379549_C/T (rs58542926)	missense	2.0 × 10^−11^ (4.1 × 10^−12^)	−0.29 (0.04)	0.054
Pro	Proline	*PRODH*	22:18910479_C/T (rs13058335)	intron	4.8 × 10^−55^ (4.5 × 10^−31^)	0.68 (0.04)	0.063

**Table 2:** Summary of genes identified in this ExWAS.

Gene	Associated Metabolite(s)	Lead, LD, or Gene Variant ^1^	Level	Description
*A1CF*	PC aa C36:0	Lead	2	Apolipoprotein B (apo B) is a major component of low-density lipoproteins and in mammals exist in two isoforms: apoB-100 and apoB-48. The two isoforms are encoded by a single mRNA transcript. *A1CF* encodes an RNA binding protein that facilitates APOBEC1’s editing of APOB mRNA, introducing a premature stop codon that yields apoB-48, resulting in the truncated gene product known as apoB-48 [20]. ApoB-48 is produced by action of APOBEC-1 exclusively in the small intestine of humans and ApoB-48 can be found in chylomicrons synthetized in the small intestine. As expected, the present of not functional APOBEC-1 enzyme resulted in impaired circulating levels of triglycerides and cholesterol and we found that it also impacts on blood levels of several PCs, such as PC aa C36:0.
*ABCC1*	C10, C12:1, C12	Lead	2	This gene encodes for an ABC proteins that transport various molecules across extra-and intra-cellular membranes.
*ACTR6*	His	LD	3	Actin Related Protein 6. The role of this gene is not fully understood as well as its association with histidine.
*AOC1*	Putrescine	Lead	1	Amine oxidase copper containing 1 (*AOC1*) encodes a metal-binding membrane glycoprotein that oxidatively deaminates putrescine, histamine, and related compounds.
*ASPG*	Asn	Lead	1	Predicted to have lysophospholipase activity and mainly responsible to catalyze the conversion of asparagine to aspartate.
*ASRGL1*	Asn	Lead	1	Encodes the l-asparaginase enzyme responsible for the catalysis of asparagine catabolism to aspartate.
*CERS4*	SM C18:0	Gene	1	This gene encodes for the protein Ceramide synthase 4, which catalyzes the formation of ceramides via sphinganine and acyl-CoA substrates, with high selectivity on long-chains.
*COL27A1*	C10, C8	Lead	3	The gene encodes a member of the fibrillar collagen family, involved in the cartilage calcification process and the transition of cartilage to bone. Mutations on this gene are known to cause Steel Syndrome.
*ENPEP*	Asp	Lead	1	*ENPEP* encodes for glutamyl aminopeptidase that regulates central hypertension through its calcium-modulated preference to cleave N-terminal acidic residues from peptides such as angiotensin II. This protein can upregulate blood pressure by cleaving the N-terminal aspartate from angiotensin II, and can regulate blood vessel formation and enhance tumorigenesis in some tissues.
*ETFDH*	C10, C6 (C4:1-DC), C8, C10:1, C12	Lead/LD	2	This gene encodes for the Electron transfer flavoprotein (ETF) present in the mitochondria, which acts in the electron transfer for at least 9 flavins. Mutations on this gene (and other ETF genes such as *ETFA* and *ETFB*) are known to cause multiple acyl-CoA deficiency (MADD), also known as glutaric acidemia
*FADS1*	PC ae C38:3, lysoPC a C26:1	LD	2	Fatty acid desaturase enzymes regulate unsaturation of fatty acids through the introduction of double bonds into the fatty acyl chain.
*FADS2*	PC ae C38:3, lysoPC a C26:1	LD	2	Fatty acid desaturase enzymes regulate unsaturation of fatty acids through the introduction of double bonds into the fatty acyl chain.
*F12; GRK6*	Asp, Taurine	Lead	3	The human coagulation factor XII (*FXII*) is involved in the intrinsic coagulation pathway.
*GLS2*	Gln	Lead	1	The gene is responsible for encoding the glutaminase 2, an enzyme that catalyzes the conversion of glutamine to glutamate and ammonia, promoting mitochondrial respiration and ATP generation.
*GNMT*	Sarcosine	LD	1	Acts on the conversion of S-adenosyl-L-methionine (SAMe) and glycine to S-adenosyl-L-homocysteine and sarcosine. Defects in this gene are a cause of hypermethioninemia.
*JMJD1C*	Putrescine	Lead	3	Plays a central role in histone code and lysine demethylation.
*LTA4H*	His	LD	3	This gene encodes an enzyme used in the final step of the biosynthesis of leukotriene B4, a proinflammatory mediator. It is known to degrade proline-glycine-proline, biomarker for chronic obstructive pulmonary disease.
*MCCC2*	C5-OH (C3-DC-M)	Lead	2	Catalyzes the conversion of 3-methylcrotonyl-CoA to 3-methylglutaconyl-CoA, playing an important role in the catabolism of leucine and isovaleric acid. Mutations in this gene are associated with 3-methylcrotonylglycinuria.
*MTHFR*	Ser	Lead	2	Responsible for the catalysis of 5,10-methylenetetrahydrofolate to 5-methyltetrahydrofolate, involved in the remethylation of homocysteine to produce methionine and tetrahydrofolate, a substrate for serine production.
*MYRF*	PC ae C38:3, lysoPC a C26:1	Lead, LD	3	Encodes an essential transcript factor that acts on the central nervous system myelination process.
*P4HA2; PDLIM4*	C16:1	Lead	3	*P4HA2* gene encodes a component of prolyl 4-hydroxylase, a key enzyme in collagen synthesis.
*PEX6*	Sarcosine	Lead	3	Encodes a member of the AAA family of ATPases, which plays a direct role in peroxisomal protein import and PTS1 (peroxisomal targeting signal 1, a C-terminal tripeptide of the sequence Ser-Lys-Leu) receptor activity.
*PMCH*	Phe	Lead	3	Responsible for the generation of multiple protein products including melanin-concentrating hormone (MCH), neuropeptide-glutamic acid-isoleucine (NEI), and neuropeptide-glycine-glutamic acid (NGE). Acts on behaviors such as hunger and arousal.
*PPID*	C10:1, C12	Lead	3	Index variant associated with different carnitines and colocalized with decreased gene expression. *PPID* is a putatively novel gene.
*PRODH*	Pro	Lead	1	This protein catalysis the intermediate reaction of proline catabolism to glutamic acid and mutations on this gene are associated with hyperprolinemia type 1.
*PSMC3*	His	Lead	3	Proteasome 26S Subunit, ATPase 3 (*PSMC3*) is a multicatalytic proteinase complex.
*PYROXD2*	C16-OH	Lead	3	Predicted oxidoreductase that may play in mitochondrial organization.
*SARDH*	Sarcosine	Lead	1	This gene encodes for the sarcosine dehydrogenase enzyme that acts on the conversion of sarcosine to glycine. Mutations in this gene are the cause for sarcosinemia.
*SLC22A1*	C3, Serotonin	Lead	1	An organic cation transporter with polyspecificity, such as for histamine, epinephrine, adrenaline, noradrenaline, dopamine, spermine and spermidine, among others.
*SLC22A5*	C0, C2, C4, C16:1	Lead/LD/gene	1	An organic cation transporter with high affinity for carnitine. Mutations in this gene are the cause of systemic primary carnitine deficiency.
*SUGP1*	PC aa C34:4	LD	3	Acts in pre-mRNA splicing.
*TCHP; GIT2*	His	Lead	3	Trichoplein keratin filament binding (*TCHP*) encodes for a protein with unknown function.
*TDO2*	Trp	Gene	1	This enzyme catalyzes the first and rate-limiting step in the conversion of tryptophan into kynurenine.
*TM6SF2*	lysoPC a C20:3, PC aa C34:4	Lead	2	Regulator of liver fat metabolism this gene influences triglyceride secretion and hepatic lipid droplet content. It is associated with fatty liver disease and non-alcoholic fatty liver disease.
*TMEM258*	lysoPC a C26:1, PC ae C38:3	Lead	3	Transmembrane Protein 258 (*TMEM258*) is a component of the oligosaccharyltransferase complex controlling ER stress and intestinal inflammation.
*TMPO*	His	Lead	3	This gene encodes several proteins containing a LEM domain through an alternative splicing mechanism. These proteins are involved in gene expression, chromatin organization, replication and cell cycle control.
*UHRF1BP1L*	His	Lead	3	UHRF1 Binding Protein 1 Like (*URHF1BP1L*) has analogy with ubiquitin-like containing PHD and RING finger domains.

^1^ Method by which this gene has been associated with the metabolite. Lead: index variant was located in the gene; LD: genome-wide significant variant (*p* < 5 × 10^−8^) in the gene was in high LD (r^2^ > 0.8) with index variant; Gene: significant association with the gene in the gene-level test.

**Table 3:** Gene-trait associations significant (unconditioned) at a 3.55 × 10^−8^ threshold in the WES combined dataset, with at least two variants needed to reach significance.

Trait ID	Trait Name	Gene	Mask ^1^	*p*-Value (Conditional)	Number of Variants	Cumulative Allele Count	Number of Variants Needed to Reach Significance
Trp	Tryptophan	*TDO2*	HMI	8.9 × 10^−9^ (1.7 × 10^−8^)	6	45	3
SM C18:0	Sphingomyeline C18:0	*CERS4*	HMI	6.1 × 10^−10^ (2.7 × 10^−8^)	10	82	3
C0	Carnitine	*SLC22A5*	HMI	2.9 × 10^−10^ (4.0 × 10^−10^)	6	37	2

^1^ HMI = high-moderate impact.

## Data Availability

The data presented in this study are available upon request to the CHRIS Access Committee. Please contact the corresponding author for details.

## Supplementary Materials

The following supporting information can be downloaded at: https://www.mdpi.com/article/10.3390/metabo12070604/s1, Figure S1: ExWAS results corrected for BMI or genotyping batch (*y*-axis) plotted against ExWAS *p*-values not corrected for BMI or genotyping batch (*x*-axis). (a) −log10 *p*-values, corrected for BMI vs. not corrected for BMI. (b) like (a), restricted to range (0, 20). (c) Beta, corrected for BMI vs. not corrected for BMI. (d) −log10 *p*-values, corrected for genotyping batch vs. not corrected for genotyping batch. (e) like (d), restricted to range (0, 20). (f) Beta, corrected for genotyping batch vs. not corrected for genotyping batch; Figure S2: Heatmap of the Spearman correlation of the rank inverse normal transformed values of the 175 metabolomic traits; Figure S3: Flowchart of the Mendelian randomization analysis; Figure S4: Minus log10 *p*-value versus beta of all traits for all index variants. For the significant association (triangle), beta is plotted against *p*-value for all other metabolites (green) and the ratios (orange) and sums (purple); Figure S5: Number of conditionally significant associations per trait, split into rare (MAF ≤ 1) and common (MAF > 1) associations. Traits without any significant associations are omitted. Traits are ordered according to their class and separated by the horizontal dotted lines; Figure S6: Minus log10 *p*-value of the skato gene test, adding the variants constituting the gene test iteratively for the 16 associations that reached significance after adding only the one best single variant. In each step i on the *x*-axis, the gene test is computed using only the i variants with the smallest single variant *p*-value. Below each point, the minor allele count is given; Figure S7: Details of the three significant gene-trait associations. (a–c) Beta is plotted against single variant –log10 *p*-value for all variants that constitute the gene test for the associations TDO2—tryptophan (a), CERS4—sphingomyeline C18:0 (b), and SLC22A5—carnitine (c). The minor allele count in the WES combined set is given below or above each point. (d–f) Quantile-quantile plots of observed versus expected *p*-values for the associations TDO2—tryptophan (d), CERS4—sphingomyeline C18:0 (e), and SLC22A5—carnitine (f); Table S1: Metabolomic traits investigated in this study; Table S2: Predefined sums investigated at significant index variants; Table S3: Predefined ratios investigated at significant index variants; Table S4: Locus-trait associations conditionally or unconditionally significant at a 5.5 × 10^−9^ threshold in at the WES combined dataset, with results given for WES and WES imputed at the best variant at the locus; Table S5: Gene-trait associations significant (unconditioned) at a 3.55 × 10^−8^ threshold in WES combined, where one variant was sufficient to reach significance; Table S6: Locus-trait associations with eQTL protein-coding genes colocalized at a PP ≥ 0.8; Table S7: Outcomes tested in the MR analysis, selected from [65]; Table S8: Results from the Mendelian randomization analysis.

## Appendix A

EBI GWAS catalog data mining: The EBI GWAS catalog r2020-11-20 in GRCh38 was downloaded and restricted to genome-wide significance (*p*-value ≤ 5 × 10^−8^). The columns “disease trait” and “*p*-value text” were identified to contain the crucial information regarding trait description and were merged. The Biocrates trait names of the 35 traits with significant associations were matched against the merged catalog column and all partial matches were extracted. False mappings, e.g., carnitine–succinylcarnitine, were manually excluded, resulting in 226 retained associations for 83 traits. Genomic coordinates in GRCh37 for the identified variants were obtained from dbSNP via the dbSNP id.
