# Supplementary material for: Whole Exome Sequencing Enhanced Imputation Identifies 85 Metabolite Associations in the Alpine CHRIS Cohort
Source: Metabolites. 2022 Jun 29;12(7):604. doi: 10.3390/metabo12070604 (PMC9320943; doi:10.3390/metabo12070604)
Supplement: Supplementary file 1 [file metabolites-12-00604-s001.zip › Figure S1.pdf]

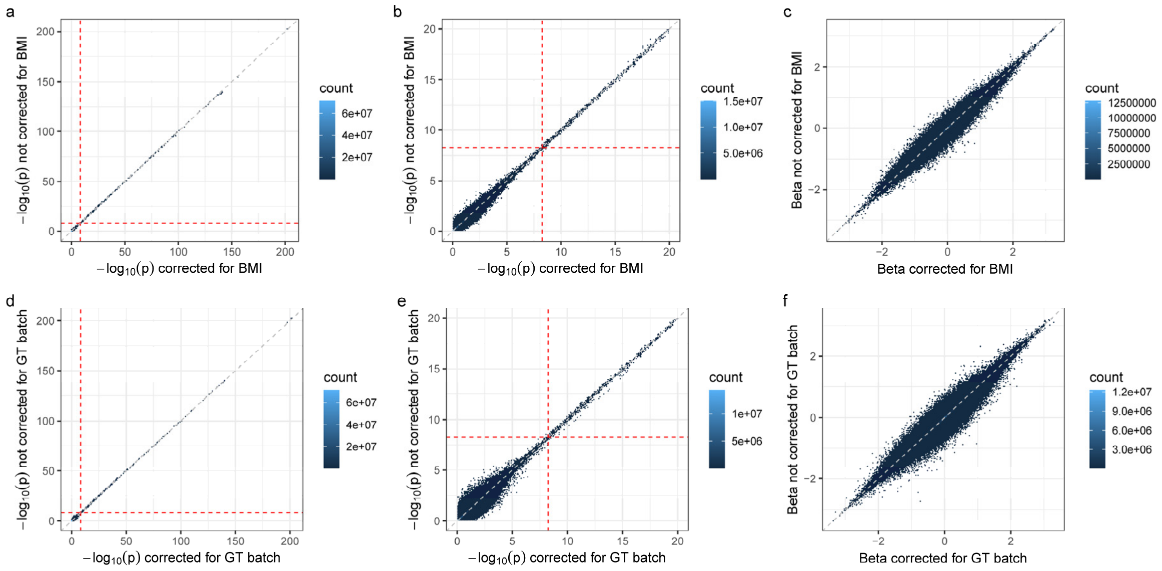

Figure S1. ExWAS results corrected for BMI or genotyping batch (*y*-axis) plotted against ExWAS *p*-values not corrected for BMI or genotyping batch (*x*-axis). (a) −log10 *p*-values, corrected for BMI vs. not corrected for BMI. (b) like (a), restricted to range (0,20). (c) Beta, corrected for BMI vs. not corrected for BMI. (d) −log10 *p*-values, corrected for genotyping batch vs. not corrected for genotyping batch. (e) like (d), restricted to range (0,20). (f) Beta, corrected for genotyping batch vs. not corrected for genotyping batch.
