# Supplementary material for: Whole Exome Sequencing Enhanced Imputation Identifies 85 Metabolite Associations in the Alpine CHRIS Cohort
Source: Metabolites. 2022 Jun 29;12(7):604. doi: 10.3390/metabo12070604 (PMC9320943; doi:10.3390/metabo12070604)
Supplement: Supplementary file 1 [file metabolites-12-00604-s001.zip › Figure S2.pdf]

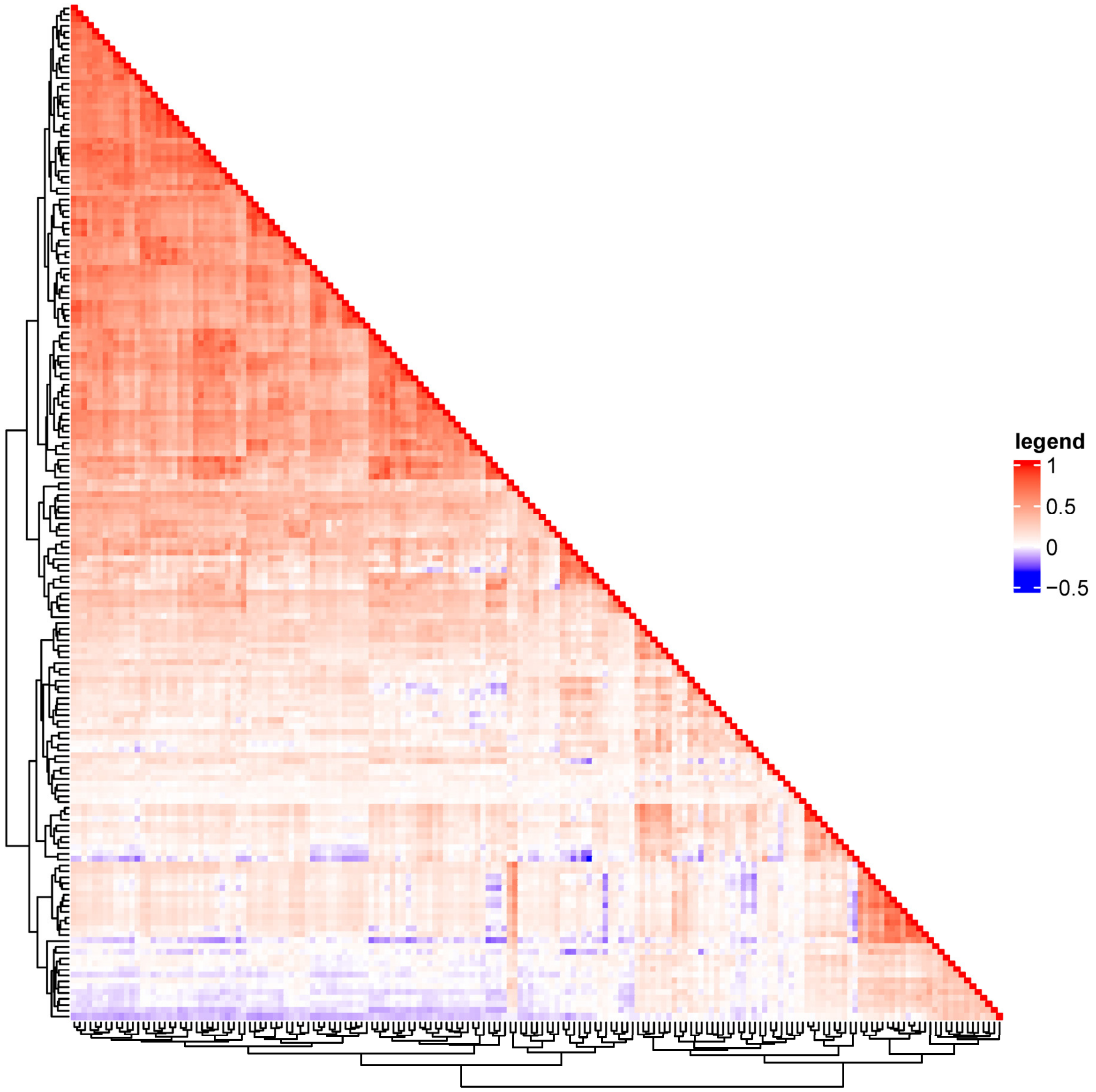

Figure S2. Heatmap of the Spearman correlation of the rank inverse normal transformed values of the 175 metabolomic traits.
