# Supplementary material for: Whole Exome Sequencing Enhanced Imputation Identifies 85 Metabolite Associations in the Alpine CHRIS Cohort
Source: Metabolites. 2022 Jun 29;12(7):604. doi: 10.3390/metabo12070604 (PMC9320943; doi:10.3390/metabo12070604)
Supplement: Supplementary file 1 [file metabolites-12-00604-s001.zip › Figure S4.pdf]

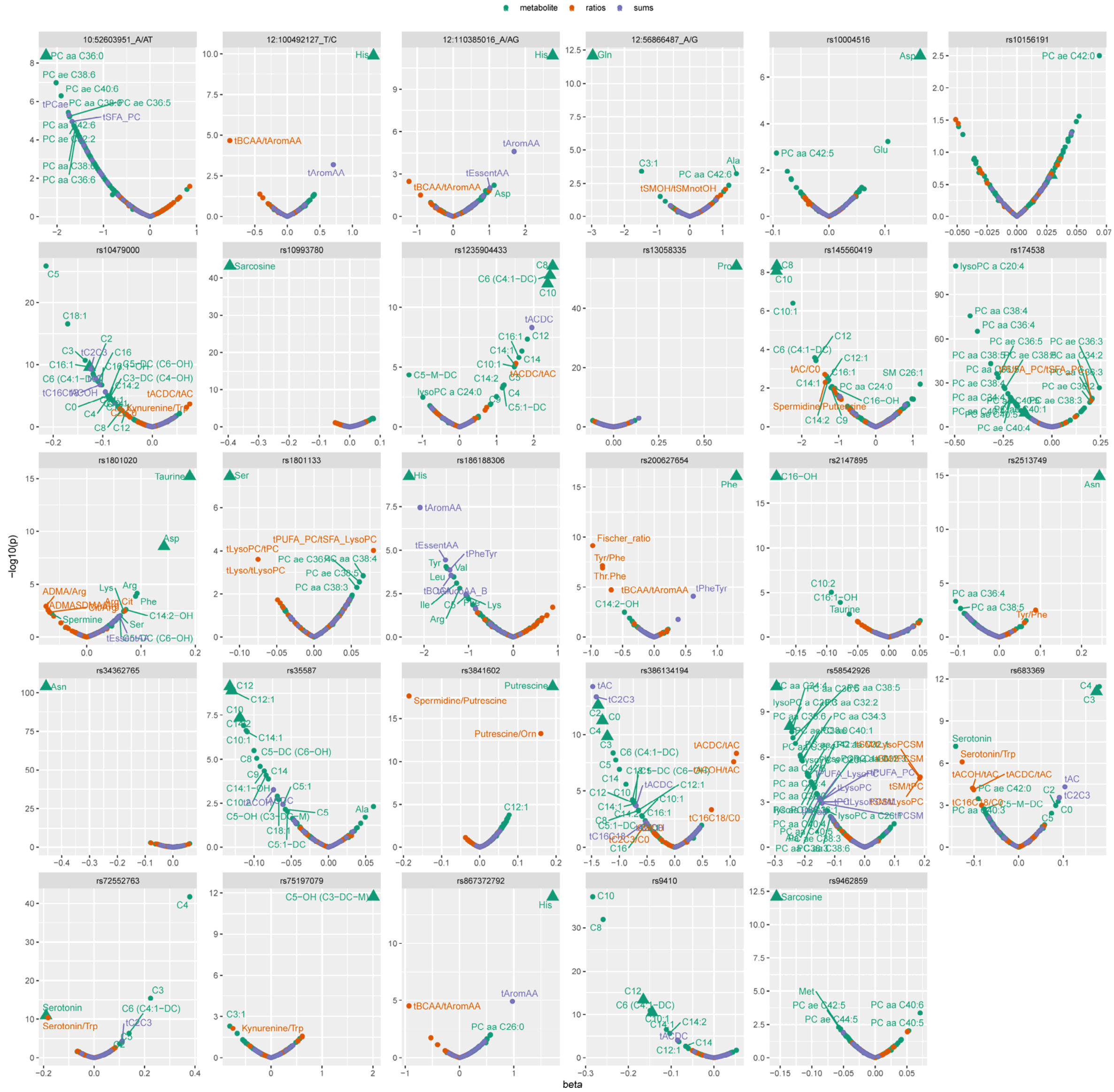

Figure S4. Minus log10 p-value versus beta of all traits for all index variants. For the significant association (triangle), beta is plotted against p-value for all other metabolites (green) and the ratios (orange) and sums (purple).
