# Supplementary material for: Whole Exome Sequencing Enhanced Imputation Identifies 85 Metabolite Associations in the Alpine CHRIS Cohort
Source: Metabolites. 2022 Jun 29;12(7):604. doi: 10.3390/metabo12070604 (PMC9320943; doi:10.3390/metabo12070604)
Supplement: Supplementary file 1 [file metabolites-12-00604-s001.zip › Figure S5.pdf]

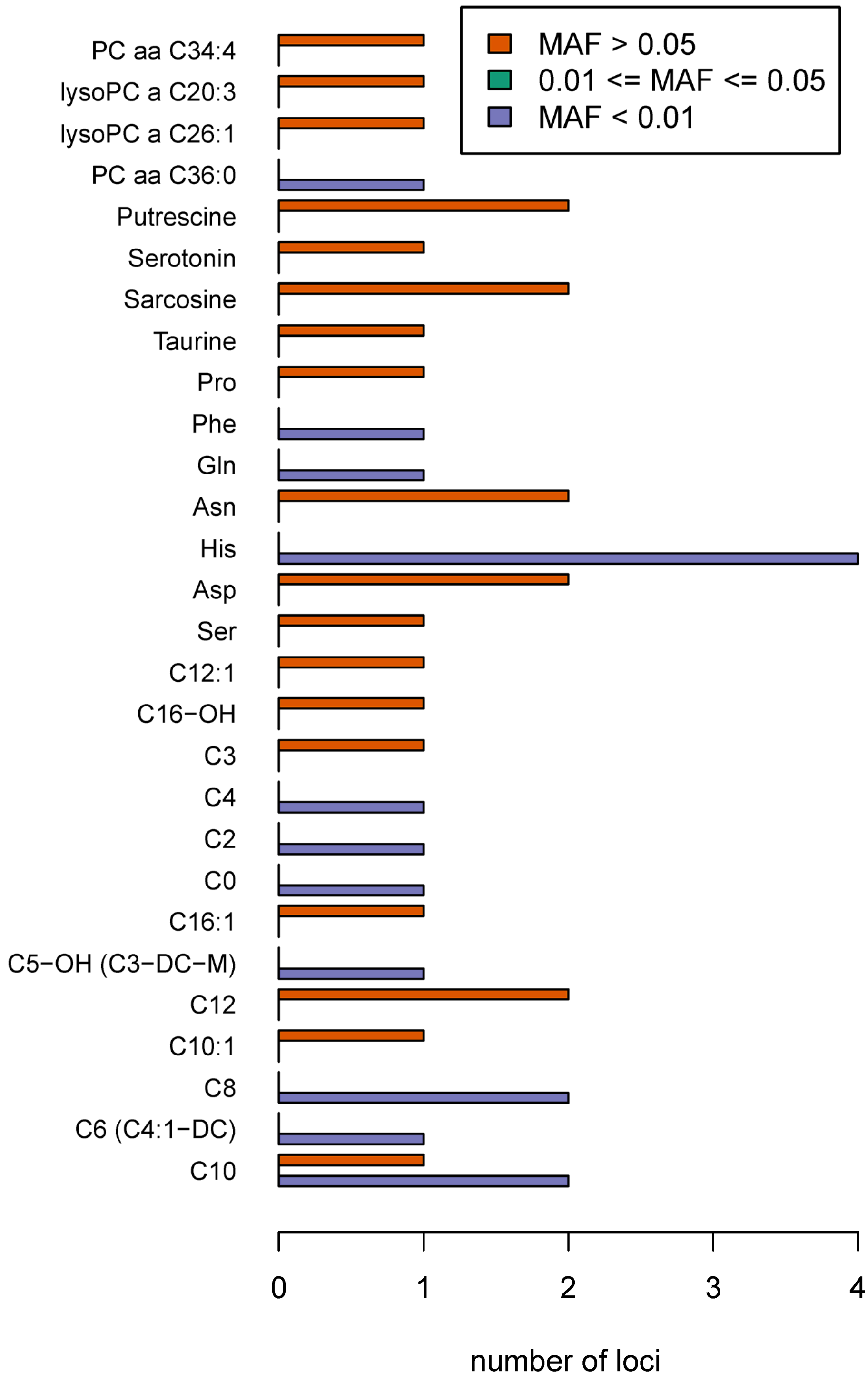

Figure S5. Number of conditionally significant associations per trait, split into rare (MAF ≤ 1) and common (MAF > 1) associations. Traits without any significant associations are omitted. Traits are ordered according to their class and separated by the horizontal dotted lines.
