# Supplementary material for: Whole Exome Sequencing Enhanced Imputation Identifies 85 Metabolite Associations in the Alpine CHRIS Cohort
Source: Metabolites. 2022 Jun 29;12(7):604. doi: 10.3390/metabo12070604 (PMC9320943; doi:10.3390/metabo12070604)
Supplement: Supplementary file 1 [file metabolites-12-00604-s001.zip › Figure S6.pdf]

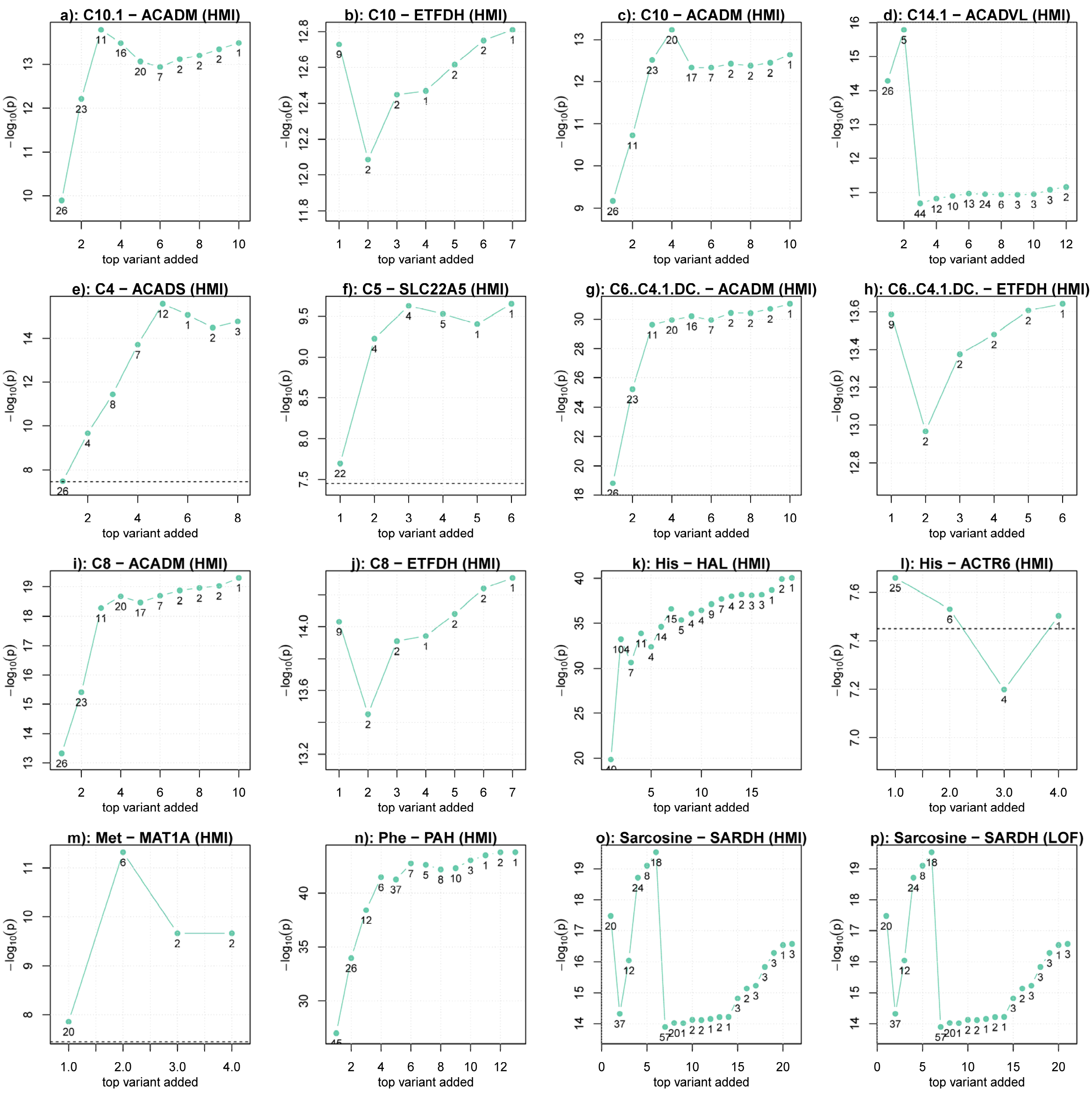

Figure S6. Minus log10 p-value of the skato gene test, adding the variants constituting the gene test iteratively for the 16 associations that reached significance after adding only the one best single variant. In each step i on the x-axis, the gene test is computed using only the i variants with the smallest single variant p-value. Below each point, the minor allele count is given.
