# Supplementary material for: Whole Exome Sequencing Enhanced Imputation Identifies 85 Metabolite Associations in the Alpine CHRIS Cohort
Source: Metabolites. 2022 Jun 29;12(7):604. doi: 10.3390/metabo12070604 (PMC9320943; doi:10.3390/metabo12070604)
Supplement: Supplementary file 1 [file metabolites-12-00604-s001.zip › Figure S7.pdf]

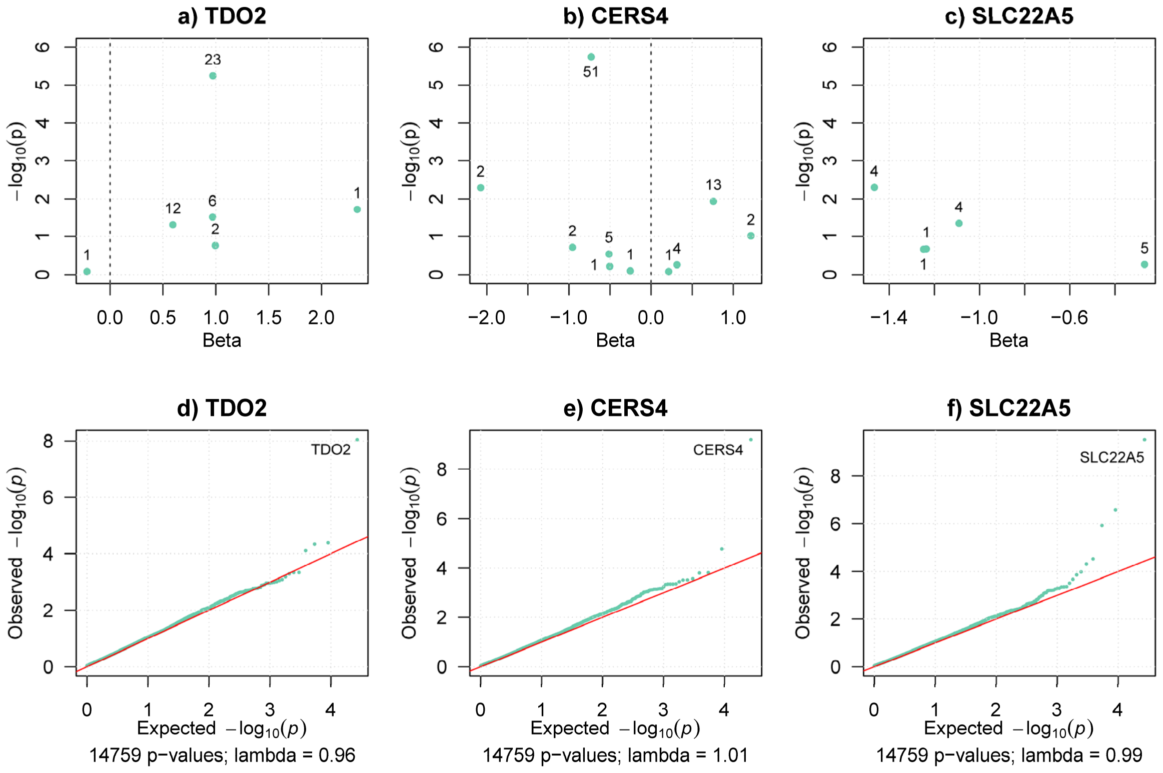

Figure S7. Details of the three significant gene-trait associations. (a) to (c) Beta is plotted against single variant –log10 *p*-value for all variants that constitute the gene test for the associations *TDO2*—tryptophan (a), *CERS4*—sphingomyeline C18:0 (b), and *SLC22A5*—carnitine (c). The minor allele count in the WES combined set is given below or above each point. (d) to (f) Quantile-quantile plots of observed versus expected *p*-values for the associations *TDO2*—tryptophan (d), *CERS4*—sphingomyeline C18:0 (e), and *SLC22A5*—carnitine (f).
